# Cytotoxicity and Antiproliferative Activity Assay of Clove Mistletoe (*Dendrophthoe pentandra* (L.) Miq.) Leaves Extracts

**DOI:** 10.1155/2016/3242698

**Published:** 2016-03-23

**Authors:** Vida Elsyana, Maria Bintang, Bambang Pontjo Priosoeryanto

**Affiliations:** ^1^Department of Biochemistry, Faculty of Mathematics and Natural Sciences, Bogor Agricultural University, Bogor 16680, Indonesia; ^2^Department of Veterinary Clinic, Reproduction and Pathology, Faculty of Veterinary Medicine, Bogor Agricultural University, Bogor 16680, Indonesia

## Abstract

Clove mistletoe (*Dendrophthoe pentandra *(L.) Miq.) is a semiparasitic plant that belongs to Loranthaceae family. Clove mistletoe was traditionally used for cancer treatment in Indonesia. In the present study, we examined cytotoxicity of clove mistletoe leaves extracts against brine shrimps and conducted their antiproliferative activity on K562 (human chronic myelogenous leukemia) and MCM-B2 (canine benign mixed mammary) cancer cell lines in vitro. The tested samples were water extract, ethanol extract, ethanol fraction, ethyl acetate fraction, and n-hexane fraction. Cytotoxicity was screened using Brine Shrimp Lethality Test (BSLT). Antiproliferative activity was conducted using Trypan Blue Dye Method and cells were counted using haemocytometer. The results showed that n-hexane fraction exhibited significant cytotoxicity with LC_50_ value of 55.31 *μ*g/mL. The n-hexane fraction was then considered for further examination. The n-hexane fraction of clove mistletoe could inhibit growth of K562 and MCM-B2 cancer cell lines in vitro. The inhibition activity of clove mistletoe n-hexane fraction at concentration of 125 *μ*g/mL on K562 cancer cell lines was 38.69%, while on MCM-B2 it was 41.5%. Therefore, it was suggested that clove mistletoe had potential natural anticancer activity.

## 1. Introduction

*Dendrophthoe pentandra *(L.) Miq. is known as mistletoe and belongs to the Loranthaceae family. In Indonesia, mistletoe is commonly known as “benalu” and it is named depending on its host plant, for example, “benalu teh” (mistletoe that grew on tea as host plant). Since* D. pentandra* is a semiparasitic plant, it reduces productivity of horticultural plants. Nevertheless,* D. pentandra* was used traditionally for cough, diabetes, hypertension, cancer, diuretic, smallpox, ulcer, skin infection, and after child-birth treatments in Indonesia and other countries [1].* D. pentandra *live on host plants to obtain food, water, and support by connecting to the host xylem via haustorium [2]. Thus, bioactivities of* D. pentandra* could also depend on its host plants where it grew [3].

Decoction of* D. pentandra *was used as complementary/alternative medicine for cancer treatment by Indonesian and Malay communities [4].* D. pentandra *that grew on various host plants were reported to have cytotoxic and anticancer activities. Daniel et al. [5] reported that ethyl acetate fraction of* D. pentandra *on soursop as host plant had cytotoxic activity against brine shrimp. Gamal and Septananda [6] revealed that* D. pentandra *growing on cottonwood decreased mutant p53 protein expression in HeLa cells in vitro. Tristanti et al. [7] studied the hepatoprotective effect of ethanol leaves extract of* D. pentandra *growing on langsat on wistar albino rats induced by carbon tetrachloride and suggested that this plant had hepatoprotective activity.* D. pentandra *growing on mango showed an ability to recover goblet cells and to repair abnormalities in colon tissue (dysplasia) [8].* D. pentandra *extract induced proliferation of mice splenocyte and thymocytes in a time- and dose-dependent manner [9].

There were several reports on bioactivities and phytochemical of mistletoe that grows on clove (clove mistletoe). Clove mistletoe leaves were found to have free radical scavenging activities [10]. Phytochemical screening of clove mistletoe leaves contained flavonoids, saponins, tannins, and triterpenoids compounds [11]. Flavonoid compounds are known to have an important role in cancer prevention [12]. However, cytotoxic and anticancer activities of clove mistletoe are still rare and some reports would be useful to provide information on plants that contain anticancer compounds.

In the present study, we examined cytotoxic activity of clove mistletoe leaves extracts against brine shrimps and their antiproliferative activity on K562 (human chronic myelogenous leukemia) and MCM-B2 (canine benign mixed mammary) cancer cell lines in vitro. The K562 and MCM-B2 cells are frequently used as model in the study of in vitro antiproliferative activity [13–18]. Clove mistletoe leaves extract would be expected to have cytotoxic activity against brine shrimps and inhibited proliferation of cancer cells.

## 2. Material and Methods

### 2.1. Plants Materials

The fresh leaves of* Dendrophthoe pentandra* (L.) Miq. from clove as host plant were collected from Lampung, Indonesia, in January, 2015. The identity of sample was authenticated from Herbarium Bogoriense, Research Centre for Biology in Indonesian Institute of Science, Bogor, Indonesia.

### 2.2. Extraction

Leaves of* D. pentandra *were firstly sun dried for 3 days and were further dried in an oven at 40–45°C for 48 h. The dried leaves were coarsely powdered using mechanical grinder. The powdered leaves extracted by water using reflux method for water extract and by ethanol 70% using maceration for ethanol extract. Ethanol extract was then fractionated using liquid-liquid extraction and resulted in ethanol fraction, ethyl acetate fraction, and n-hexane fraction. Each of the extracts and fractions were evaporated by vacuum evaporator.

### 2.3. Brine Shrimp Lethality Test (BSLT)

The cytotoxic activity of extracts and fractions was performed using BSLT method described by McLaughlin et al. [19] with slight modification. Brine shrimps (*Artemia salina* Leach) larvae were used as test organisms. The cysts were placed and hatched for 48 hours with constant oxygen supply and under the light at room temperature. Twenty milligrams of the extract was added to the test tube containing 50 *μ*L Tween80 in 10 mL of sea water to get stock solution (2000 *μ*g/mL). The four graded sample concentrations (1000, 500, 100, and 10 *μ*g/mL) were used in the experiment. The experiments were done in triplicate. A test tube containing 50 *μ*L Tween80 in 10 mL of sea water and 10 living brine shrimps was used as the negative control. The surviving larvae were counted after 24 hours of samples exposure. Larvae were considered dead if they did not exhibit any movement during 10 seconds of observation. Sample was considered toxic to brine shrimps if it had LC_50_ (lethal concentration 50%) less than 1000 *μ*g/mL [20]. The data of surviving brine shrimp was analyzed with SPSS 16.0 program for probit analysis to determine LC_50_ values and 95% confidence intervals.

### 2.4. Antiproliferative Activity Assay

Antiproliferative activity assay of extracts and fractions was conducted according to the method of Priosoeryanto et al. [21]. In vitro antiproliferative activity was assessed with K562 (human chronic myelogenous leukemia) and MCM-B2 (canine benign mixed mammary) cancer cell lines. The K562 and MCM-B2 cells were obtained from Laboratory of Tissue Culture, Department of Veterinary Clinic, Reproduction and Pathology, Faculty of Veterinary Medicine, Bogor Agricultural University, Indonesia. The cell lines were cultured in the tissue culture 24-well plates at a density of 7 × 10^5^ cells/mL. These cells were maintained in Dulbecco's Modified Eagle's Medium (DMEM), supplemented with 10% fetal calf serum, antibiotics (penicillin and streptomycin), and fungizone as antifungal. The extracts were dissolved in the tube containing 100 *μ*L of dimethyl sulfoxide (DMSO) in sterile distilled water. Each concentration of extract was added to the cultured well in triplicate. The tested concentrations of extract were 25, 50, 75, 100, and 125 *μ*g/mL. DMEM was used as negative control and doxorubicin (100 *μ*g/mL) was used as positive control. These cells were incubated in a humidified atmosphere of 5% CO_2_ in air, at 37°C. The cells were harvested when the cells growth in the negative control was confluent (±3 days). The cells were then stained using Trypan Blue Dye. The average of the total number of the cells on each well was counted under light microscope on 100x magnification using Improved Neubauer Haemocytometer.

### 2.5. Statistical Analysis

All analytical values shown represent the means of three replicates. Data of cell proliferation inhibition were analyzed using one-way ANOVA by SPSS 16.0 (Statistical Package for the Social Sciences) for Windows. Mean separation test between treatments was performed using Duncan's multiple range test. *P* value ≤ 0.05 was considered statistically significant.

## 3. Results and Discussion

In the drug development process, a large number of crude plant-derived extracts were firstly screened for their cytotoxic activity before they were further assayed using cancer cell lines or higher animals. Many researches showed that Brine Shrimp Lethality Test (BSLT) has a good correlation with various tumour cell lines. The cytotoxic activity of extract in BSLT is determined by 50% brine shrimps mortality response (LC_50_). The plant extract was considered toxic to brine shrimps if it had LC_50_ < 1000 *μ*g/mL [20]. The toxicity level would show potency of extracts as anticancer.

In the present study, the LC_50_ values of* D. pentandra* leaves extracts and fractions have been shown in Table 1. Water extract, ethanol extract, and ethanol fraction of* D. pentandra* growing on clove had LC_50_ value greater than 1000 *μ*g/mL. The water extract showed no cytotoxic activity against brine shrimp. This result was in accordance with the water extracts of* D. pentandra *growing on* Annona squamosa, Camellia sinensis, Spondias dulcis, *and* Stelechocarpus burahol* (LC_50_ > 1000 *μ*g/mL) reported by Artanti et al. [1]. But this result was different from previous studies which reported that* D. pentandra* water extract from Kelantan, Malaysia, had cytotoxic activity against brine shrimps (LC_50_ 251.22 *μ*g/mL) [4]. Since BSLT was used for preliminary screening for detection of anticancer compounds, this result showed that the use of* D. pentandra* decoction was less effective for cancer treatment. Nevertheless, water extract is still relatively safe to be consumed as antioxidant for cancer chemoprevention. Fitrilia et al. [11] reported that water extract of clove mistletoe had DPPH free radical scavenging activity with IC_50_ value of 11.4 *μ*g/mL. This high antioxidant activity was not significantly different with *α*-tocopherol (IC_50_ 6.08 *μ*g/mL). Previous research also reported that aqueous extract of* D. pentandra* had high antioxidant with IC_50_ value of 0.1% [22].

The clove mistletoe ethanol extract also showed no cytotoxic activity according to BSLT method (Table 1). But Daniel et al. [5] reported that ethanol extract of* D. pentandra* growing on soursop had cytotoxic activity with LC_50_ value of 4.53 *μ*g/mL. Nevertheless, the clove mistletoe ethanol extract had high antioxidant activity with IC_50_ value of 6.8 *μ*g/mL [11]. Widowati et al. [23] reported that* D. pentandra* ethanol extract showed DPPH free radical scavenging activity (IC_50_ 4.74 *μ*g/mL) and it was comparable with ascorbic acid and quercetin (IC_50_ 2.16 and 3.24 *μ*g/mL, resp.).

The data in Table 1 showed that ethanol fraction of* D. pentandra* had no cytotoxic activity against brine shrimps. This result was validated with previous research which showed that ethanol fraction of* D. pentandra* was nontoxic on male and female mice (LD_50_ > 2000 mg/Kg body weight) [24], but methanol fraction of* D. pentandra* was toxic towards brine shrimp [4, 5].

The ethyl acetate and n-hexane fractions of* D. pentandra* exhibited cytotoxic activity against brine shrimps (Table 1). The ethyl acetate and n-hexane fractions could kill 50% of brine shrimps at concentration less than 1000 *μ*g/mL (Table 1). It indicated that ethyl acetate and n-hexane fractions exhibit toxicity against brine shrimps. This result suggested that ethyl acetate and n-hexane fractions had potent anticancer activity in cancer cell lines. Nevertheless, n-hexane fraction was eleven times more potent than ethyl acetate fraction. Therefore, only n-hexane fraction was then considered to perform in antiproliferative activity assay in vitro.

The cytotoxic activity of ethyl acetate and n-hexane fractions of* D. pentandra* against brine shrimps was also reported by Daniel et al. [5]. The ethyl acetate fraction of* D. pentandra* on soursop as host plant had LC_50_ value of 2.13 *μ*g/mL, while n-hexane fraction had LC_50_ value of 3305.04 *μ*g/mL. These results suggest that cytotoxic activity of mistletoe depends on its host plants and extraction solvent [1, 3].

Brine shrimps mortality after n-hexane fraction exposure was expected to be associated with bioactive compound and not with starvation. This was confirmed by the absence of brine shrimps death in negative control group. Fitrilia et al. [11] reported that clove mistletoe leaves extract contained flavonoid and triterpenoids. Flavonoids and triterpenoids were toxic for brine shrimps in a concentration-dependent manner, so it might have contributed to brine shrimps mortality.

The n-hexane fraction was reported to have the lowest antioxidant activity [11], but it exhibited the highest cytotoxic activity compared to other extracts and fractions that also contained flavonoid and triterpenoids. We assumed that the n-hexane fraction might contain other active compounds or other constituents might have had a synergistic effect on cytotoxic activity [24, 25].

**Figure 1:**
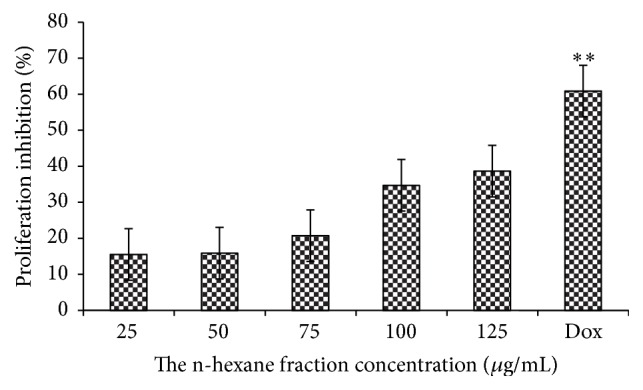
Percentage of proliferation inhibition K562 cells after treatment with n-hexane fraction of clove mistletoe. The data is expressed as percentage of proliferation inhibition ± SEM, as compared to the negative control (100%). Level of significance is denoted as follows: ^*∗∗*^ *P* < 0.01.

**Figure 2:**
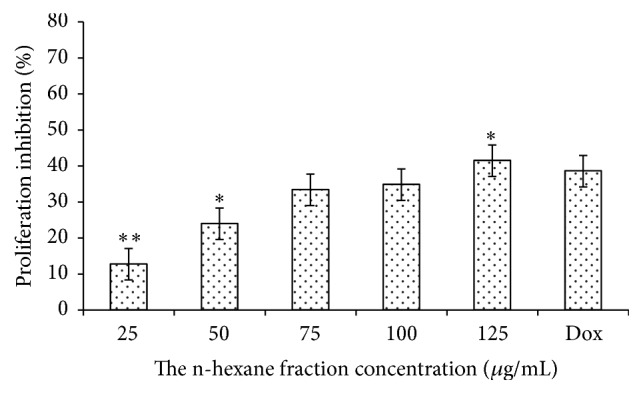
Percentage of proliferation inhibition MCM-B2 cells after treatment with n-hexane fraction of clove mistletoe. The data is expressed as percentage of proliferation inhibition ± SEM, as compared to the negative control (100%). Level of significance is denoted as follows: ^*∗*^ *P* < 0.05; ^*∗∗*^ *P* < 0.01.

The antiproliferative activity in vitro of n-hexane fraction was conducted with K562 (human chronic myelogenous leukemia) and MCM-B2 (canine benign mixed mammary) cancer cell lines. The K562 and MCM-B2 proliferation inhibitions after n-hexane fraction treatment were presented in Figures 1 and 2. K562 and MCM-B2 cancer cells decreased with the increase of the given tested concentrations. At the lowest tested concentration (25 *μ*g/mL), n-hexane fraction could inhibit cell of both cancer cell lines.

The n-hexane fraction could inhibit more than one-third of K562 and MCM-B2 cells at the tested concentration of 125 *μ*g/mL. Inhibition activity of n-hexane fraction showed different effect on both cancer cell lines. Inhibition activity of n-hexane fraction on MCM-B2 cells was slightly greater than K562 and commercial drug (doxorubicin). This is in accordance with Priosoeryanto et al. [26] who reported that anticancer activity from plants extracts depends on cancer cell lines that were used in experiment.

Previous study reported that ethanol extract of* D. pentandra* had very low antiproliferative activity in T47D cells with IC_50_ value of 728.05 *μ*g/mL [27]. But* D. pentandra* aqueous extract could inhibit T47D cells proliferation with IC_50_ value of 1.2% and induce apoptosis [23]. Zainuddin and Sul'ain [28] also reported that* D. pentandra* ethyl acetate extract was effective towards MCF-7 breast cancer cell line with IC_50_ value of 14.42 ± 0.34 *μ*g/mL, while methanol extract had IC_50_ value of 17.70 ± 0.21 *μ*g/mL.* D. pentandra* leaves extract had no cytotoxic activity on mammalian normal cell lines (MDCK, L929, and Vero) [4, 28]. This suggests that* D. pentandra* had potency for cancer treatment and is relatively nontoxic and safe for traditional/complementary medicine.

The K562 cancer cell line is blast cell that was characterized by highly undifferentiated and difficult to mature functional cells [29]. Priosoeryanto et al. [30] reported that MCM-B2 might be derived from stem cells or atypical cells, so its cells were undifferentiated. Observation in vitro of MCM-B2 cell line shows that these cells exhibit homogeneously morphological and immunohistochemical characteristics. MCM-B2 also shows less differentiation in cell culture. This undifferentiated character of K56 and MCM-B2 might contribute to inhibition of both cells growth when treated using n-hexane fraction.

Treatment of K562 and MCM-B2 cells with n-hexane fraction of clove mistletoe resulted in different proliferation inhibition activity. It might be associated with membrane and nuclear receptors on K562 and MCM-B2 cells. Different receptors on K562 and MCM-B2 cells may have a different response for active compounds of clove mistletoe n-hexane fraction.

The n-hexane fraction of clove mistletoe previously reported the presence of flavonoid and triterpenoid [11]. Flavonoid and triterpenoids are known to have important roles in cancer chemoprevention and chemotherapy [12]. These compounds were reported to have antioxidant activity, with capacity to scavenge reactive oxygen species (ROS) [31, 32]. ROS have long been known to be cytotoxic and the cause of various diseases including cancer, diabetes, and neurodegenerative disease [33]. Anticancer mechanism of flavonoid and triterpenoids based on their antioxidant properties is associated with their ability to scavenge free radicals, inhibit the enzymes involved in ROS formation, and block the oxidation of cellular and extracellular compounds [32, 34]. Moreover, flavonoids contribute to modulating pathway of cancer proliferation, arresting the cell cycle, inducing apoptosis, and inhibiting angiogenesis [31, 35]. Therefore, our present results provide better information on clove mistletoe traditionally used for cancer treatment.

## 4. Conclusion

Clove mistletoe leaves extract, particularly n-hexane fraction, exhibited cytotoxic activity with LC_50_ value of 55.31 *μ*g/mL. The n-hexane fraction also possessed antiproliferative activity on K562 and MCM-B2 cancer cell lines. The inhibition activity of clove mistletoe n-hexane fraction at concentration 125 *μ*g/mL on K562 and MCM-B2 was 38.69% and 41.5%, respectively. Therefore, it was suggested that clove mistletoe had a potent natural anticancer activity.

## Figures and Tables

**Table 1:** Cytotoxic activity of *D. pentandra *leaves extracts and fractions against brine shrimps.

Sample	% brine shrimp mortality (concentration in *μ*g/mL)				LC_50_ (*μ*g/mL)
	10	100	500	1000	
Water extract	16.67 ± 0.58	20.00 ± 0.0	26.67 ± 0.58	30.00 ± 0.00	>1000
Ethanol extract	0.00 ± 0.00	3.33 ± 0.58	3.33 ± 0.58	16.67 ± 0.58	>1000
Ethanol fraction	0.00 ± 0.00	3.33 ± 0.58	3.33 ± 0.58	10.00 ± 0.00	>1000
Ethyl acetate fraction	3.33 ± 0.58	23.33 ± 1.15	50.00 ± 1.00	70.00 ± 1.73	649.12
n-Hexane fraction	40.00 ± 0.00	76.67 ± 0.58	100.00 ± 0.00	100.00 ± 0.00	55.32

Note: percentages of brine shrimp mortality were expressed as mean ± SD.
